# Transcriptome Mining to Identify Molecular Markers for the Diagnosis of *Staphylococcus epidermidis* Bloodstream Infections

**DOI:** 10.3390/antibiotics11111596

**Published:** 2022-11-11

**Authors:** Susana Brás, Angela França

**Affiliations:** 1 LIBRO—Laboratório de Investigação em Biofilmes Rosário Oliveira, Centre of Biological Engineering, University of Minho, Campus de Gualtar, 4710-057 Braga, Portugal; 2 LABBELS–Associate Laboratory, Braga and Guimarães, Portugal

**Keywords:** bloodstream infection diagnosis, commensal isolates, clinical isolates, ex vivo human blood model, RNA sequencing, molecular diagnosis markers

## Abstract

Bloodstream infections caused by *Staphylococcus epidermidis* are often misdiagnosed since no diagnostic marker found so far can unequivocally discriminate “true” infection from sample contamination. While attempts have been made to find genomic and/or phenotypic differences between invasive and commensal isolates, possible changes in the transcriptome of these isolates under in vivo-mimicking conditions have not been investigated. Herein, we characterized the transcriptome, by RNA sequencing, of three clinical and three commensal isolates after 2 h of exposure to whole human blood. Bioinformatics analysis was used to rank the genes with the highest potential to distinguish invasive from commensal isolates and among the ten genes identified as candidates, the gene *SERP2441* showed the highest potential. A collection of 56 clinical and commensal isolates was then used to validate, by quantitative PCR, the discriminative power of the selected genes. A significant variation was observed among isolates, and the discriminative power of the selected genes was lost, undermining their potential use as markers. Nevertheless, future studies should include an RNA sequencing characterization of a larger collection of isolates, as well as a wider range of conditions to increase the chances of finding further candidate markers for the diagnosis of bloodstream infections caused by *S. epidermidis*.

## 1. Introduction

*Staphylococcus epidermidis* is a commensal bacterium of healthy human skin and mucosae. However, it has become one of the most common causes of medical device-related infections, being a frequent source of catheter-related bloodstream infections [1]. Diagnosing bloodstream infections caused by *S. epidermidis* is a daily challenge for clinicians, as due to its commensal nature it is difficult to discriminate between blood culture contamination and infection [2]. This often results in misdiagnosis, which consequently leads to increased mortality and morbidity rates, along with additional healthcare costs due to prolonged hospital stays and further testing and treatments [3]. Therefore, an accurate diagnosis is essential to reduce the clinical and economic burden associated with medical device-related bloodstream infections caused by *S. epidermidis*. Over the last years, several studies have attempted to identify markers capable of discriminating infection from blood culture contamination, presumably caused by commensal isolates. These efforts have been mainly focused on phenotypic- or DNA-based strategies. A whole genome sequencing analysis of several *S. epidermidis* isolates has shown that the genomes of the population could be separated into two phylogenetic groups: lineage A/C, which contained most of the isolates recovered from colonization and infection and lineage B, consisting only of commensal isolates [4]. Later, it was demonstrated that these lineages have different pathogenic potentials, with the lineage A/C showing a higher adaptation ability in infection-like conditions [5]. It has also been shown, among others, that the presence of the genes *ica*, *bhp*, *sesI,* and *sdrF*, in addition to the carriage of the insertion sequence 256 and SCCmec elements, are often associated with clinical isolates. In contrast, commensal isolates are often positive to the genes *aap* and *fdh,* and the arginine catabolic mobile element [4,5,6,7,8,9]. Furthermore, it was recently discovered that the genes *tarIJLM* are absent in commensal isolates [10]. However, only healthcare-associated methicillin-resistant *S. epidermidis* strains seem to harbor these genes [10], hindering their use as diagnostic markers. Finally, there have also been efforts to improve diagnostic accuracy by combining phenotypic markers and the presence of specific virulence genes, achieving a diagnostic accuracy of 82.4% [11]. However, despite the clear advances, no marker can rapidly and with high sensitivity distinguish infection from contamination. As such, the search for novel diagnostic markers is still urgent to diminish the clinical consequences associated with bloodstream infections caused by *S. epidermidis*.

In that sense, considering that gene expression is regulated in response to environmental conditions, which can determine the ability of *S. epidermidis* to adapt, we hypothesized that the comparative analysis of the transcriptome of clinical and commensal isolates in the context of infection could help to find suitable discriminative markers. To address this hypothesis, we investigated, using RNA sequencing (RNA-seq), the transcriptome of three clinical and three commensal isolates after interaction with whole human blood.

## 2. Results

### 2.1. Identification and Selection of Candidate Diagnostic Markers

The experimental set-up and the in silico analysis devised to identify markers with the potential to discriminate clinical from commensal isolates are depicted in Figures S1 and S2, respectively. Regarding the in silico analysis, three strategies were devised. In the first approach, genes expressed only in one of the groups of isolates were selected. While five genes were identified, the gene *SERP2441* had the higher discriminative potential. As the number of genes identified in this approach was limited, a second strategy was applied. The aim was to select highly expressed genes in both groups whose relative expression could differentiate clinical from commensal isolates. However, all identified genes had very similar expression values, failing to discriminate the origin of the isolates. Hence, a third strategy was performed to search for genes differentially expressed between both groups but using different expression level thresholds. Nine genes were underscored as potential markers among the 1947 genes identified with this last strategy. In the end, ten genes were considered possible candidates for diagnostic markers (Table S1), and their potential was further evaluated.

**Figure 1:**
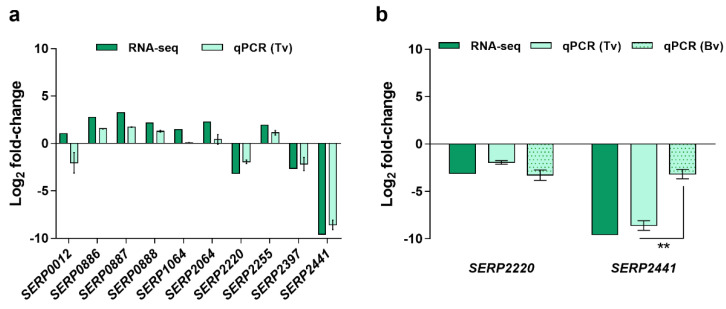
Validation of the results obtained by RNA-seq. Data are presented in fold-change expression calculated using commensal isolates as control. (**a**) Technical validation (Tv) was performed using the same RNA utilized for library construction. The bars represent the mean and the standard error of the mean of two to three technical replicates. (**b**) Biological validation (Bv) was accomplished using RNA obtained from independent experiments. These experiments were performed with the same three clinical and three commensal isolates used for RNA-seq. The bars represent the mean and the standard error of the mean of three independent experiments performed using the blood of three different donors (*n* = 3). Statistical differences between technical and biological validations (**b**) were analyzed with the unpaired *t*-test. ** *p* < 0.001. RNA-seq, RNA sequencing, qPCR, quantitative PCR.

First, RNA-seq data was validated, by quantitative PCR (qPCR), using the same RNA employed for RNA-seq (technical validation). Except for gene *SERP0012*, the same trend was obtained in both methods, although the gene *SERP1064* showed a substantially lower discriminative potential when detected by qPCR (Figure 1a). The discrepancies observed between methods may be due to artifacts created during library construction or sequencing, or the low level of expression of the genes selected, impairing the detection and the correlation between methods [12]. Considering the statistical significance of the differential expression observed in RNA-seq, as determined by Baggerley’s test with FDR correction with a cutoff of 0.05, the potential to discriminate clinical from commensal isolates, i.e., with high differential expression in both RNA-seq and qPCR, the genes *SERP2220* and *SERP2441* were selected for further analysis, namely for the biological validation assays. Genes *SERP2220* and *SERP2441* encode, respectively, a putative universal stress protein and a membrane-bound solute carrier 45 family major facilitator superfamily transporter. As can be seen in Figure 1b, even though both genes maintained the trend observed in RNA-seq, a significant decrease in the discriminative power was observed, with higher significance in the case of the gene *SERP2441*. Notwithstanding, the gene *SERP2441* was still the most promising since it presented the higher differential expression between clinical and commensal isolates (≈3 × higher than *SERP2220*) and, thus, was selected for additional validation steps. In addition, we found out, when developing an unrelated work, that the expression of the gene *SERP2220* was significantly downregulated in clinical strains (strains RP62A and 9142) when incubated in a routine laboratory medium (Tryptic Soy Broth). This indicates that the alteration observed in human blood is not specific to the condition, and, as such, *SERP2220* was excluded from our list.

### 2.2. Validation of the Candidate Diagnostic Marker

**Figure 2:**
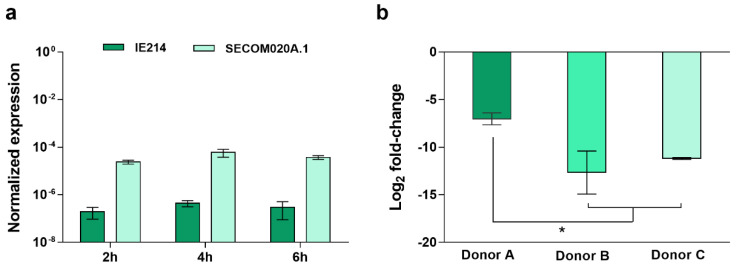
Evaluation of the discriminative potential of the gene *SERP2441*. Analysis of the gene *SERP2441* expression stability (**a**) over time (*n* = 3, using the blood of the same donor) and (**b**) 4 h upon incubation with the blood of three different donors (*n* = 2 to 3 technical qPCR replicates). These experiments were performed in strains IE214 (clinical isolate) and SECOM0020A.1 (commensal isolate). Statistical differences among groups were analyzed with one-way ANOVA and Tukey’s multiple comparison test. * *p* < 0.05.

Because of the transient nature of gene expression, to substantiate whether the difference detected in the expression of the gene *SERP2441* upon 2 h of interaction with human blood would maintain over time, we investigated its transcription stability for up to 6 h. Moreover, due to the known variability inherent to blood donors [13,14,15], we have also examined the stability of gene *SERP2441* expression in the blood of different donors (Figure 2). For these assays, one clinical and one commensal isolate, which showed a pronounced difference in the expression of the gene *SERP2441*, were used.

As can be seen in Figure 2a, no significant alterations were found among the different time points. In contrast, the expression of the gene *SERP2441* varied when incubated with the blood of different donors. However, despite the differences found, the same tendency was observed, and the potential to differentiate clinical from commensal isolates were maintained (Figure 2b). Additionally, bearing in mind the recognized diversity within the *S. epidermidis* population [16], the expression of the target gene was investigated in a larger collection of isolates. As such, 86 isolates, composed of 38 isolates from infection, 24 from the skin of healthy individuals with no contact with the hospital environment, and six that were culture contaminants or isolates from the skin of hospitalized patients and staff (designated as “contamination” from now on) were first tested, by PCR, for the presence of the gene *SERP2441*. While all isolates from the commensal and contamination groups harbored the gene *SERP2441*, only 26 of the 38 clinical isolates tested were positive for the gene (Table S2). Consequently, only the 56 isolates positive for the gene *SERP2441* were used further from this collection.

**Figure 3:**
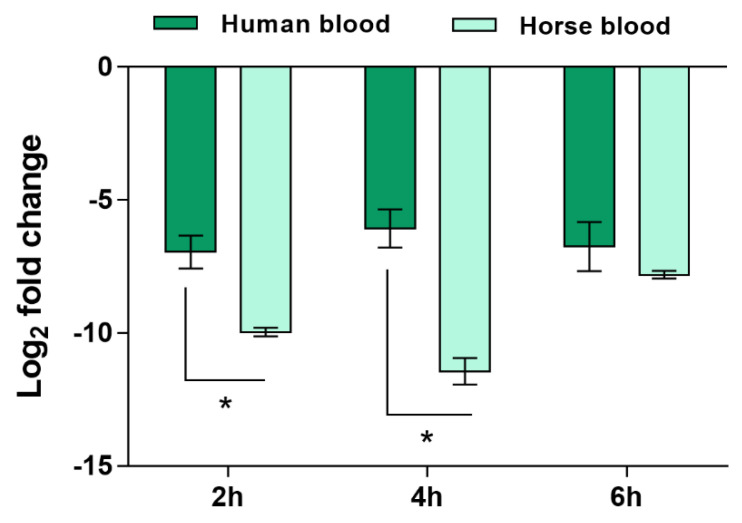
Evaluation of the expression of the gene *SERP2441* in human and defibrinated horse blood over time. The bars represent the mean and the standard error of the mean of two independent assays (*n* = 2). These experiments were performed in strains IE214 (clinical isolate) and SECOM0020A.1 (commensal isolate). Statistical differences between groups in each time point were analyzed with the unpaired *t*-test. * *p* < 0.05.

As can be anticipated, a high quantity of human blood would be necessary to test the expression levels of *SERP2441* in the 56 isolates. To decrease the use of human blood and considering that we have previously demonstrated that blood soluble factors, and not the cells, strongly affect gene expression of *S. epidermidis* [17], we hypothesized that the utilization of commercially available defibrinated mammal blood could be an alternative. Thus, to test this hypothesis, we compared the expression levels of *SERP2441* in human and defibrinated horse blood, for up to 6 h, in one clinical and one commensal isolate. Despite the slightly higher differential expression in horse blood observed in the first 4 h of interaction, the same trend was obtained in both types of blood (Figure 3). Henceforth, the expression level of *SERP2441* in the collection of 56 isolates was determined using horse blood.

**Figure 4:**
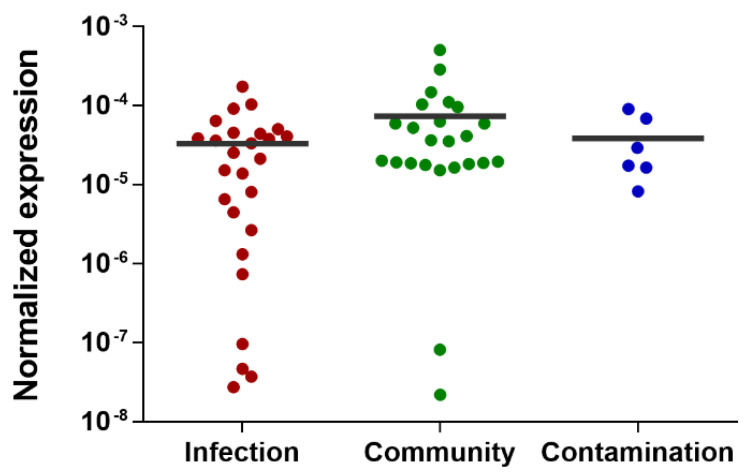
Evaluation of the discriminative potential of the gene *SERP2441* in a wide collection of isolates. This evaluation was performed 2 h after incubation with horse blood in a collection of 56 isolates. Of note, the isolates belonging to the “contamination” group are isolates collected from either contaminated samples or the skin of patients or staff. The horizontal line represents the grand mean of the transcription level of all isolates (*n* = 2 to 3 technical qPCR replicates). Statistical differences among groups were analyzed with one-way ANOVA and Tukey´s multiple comparison test.

Unfortunately, the expression of the gene *SERP2441* was highly variable among the isolates tested, with some strains expressing *SERP2441* approximately 23.000-fold more than other isolates within the same group. This resulted in the loss of gene *SERP2441* discriminative potential (Figure 4). Hence, we tested other candidates, although with lower potential, namely the genes *SERP0887*, *SERP1064*, and *SERP2064*, which encode an ABC transporter permease, a short-chain oxidoreductase dehydrogenase and a protein from the type 2 phosphatidic acid phosphatase family. These genes were selected based on either the statistical significance of the differential expression obtained by RNA-seq (Baggerley’s test with FDR correction with a cutoff of 0.05), the level of differential expression, or the consistency of the results obtained in the technical validation by qPCR. Of note, the presence of these genes in the genome of all strains (Table S2) and their expression level in human and horse blood (Figure S3) were also previously confirmed. Again, no significant differences were detected among the groups tested (Figure S4).

## 3. Discussion

Considering that *S. epidermidis* virulence factors seem to be the same that confer its fitness as a commensal [1], we hypothesized that the ability of *S. epidermidis* to adapt to the host environment may not depend on a specific phenotypic and/or genetic makeup, but rather on the regulation of gene transcription. In addition, it was recently shown that the expression of the protease EcpA aggravates the clinical symptoms in patients with atopic dermatitis [18], further supporting the importance of gene expression regulation in the ability of *S. epidermidis* to cause disease. Therefore, we have sequenced the transcriptome of commensal and clinical isolates upon interaction with human blood to identify different regulatory mechanisms between isolates.

Among the genes with differential expression between the clinical and commensal isolates, the gene *SERP2441* was the one with higher discriminative potential. As only three clinical and three commensal isolates were analyzed by RNA-seq, to validate the results obtained, a larger collection of isolates was tested by quantitative PCR. Unfortunately, high variability was observed in the expression of *SERP2441* among the isolates of the same group, resulting in the loss of discriminative power. Similarly, due to the high variability among *S. epidermidis* isolates, Both and colleagues could not detect significant differences in the transcriptome of ten clinical and ten nasal commensal isolates of *S. epidermidis* isolated from five different patients in the presence of 50% of human plasma [19]. Together, our data suggest no distinguishing transcriptomic pattern among isolates. However, it is important to acknowledge that due to the low number of isolates analyzed by RNA-seq, the identification of potential markers may have been biased. Indeed, the three clinical isolates selected for RNA-seq analysis seem to have an atypical absence of expression of the gene *SERP2441*, which may biased our analysis. Thus, we cannot exclude the possibility that by sequencing the transcriptome of a larger collection of isolates, as well as more time points, novel and more reliable potential markers could have been identified. Moreover, as Teichmann et al. have recently demonstrated, the expression of a particular set of genes is dependent on the isolation place (skin vs. nose) [20]. Since, in our study, we have used an array of isolates collected from a variety of host niches and diverse geographic and clinical origins, we speculate that this diversity may have also influenced our results. Furthermore, it was recently shown that the diversity in the *S. epidermidis* population might be related to the fact that different human body niches are colonized by different clonal lineages of *S. epidermidis* [21]. As we do not have molecular typing data for all isolates, we could not determine if the isolates tested herein are genetically related, constituting another limitation of this study.

Overall, under our conditions, the transcriptome-based approach was not able to differentiate clinical from commensal isolates as initially hypothesized. Nevertheless, we speculate that this may be related to the limitations of our study. As such, this approach shall be considered in a larger number of isolates, together with a complete typing characterization, and under a more diversified range of conditions. Nevertheless, to the best of our knowledge, this is the first study to investigate the transcriptome of clinical and commensal isolates in human blood. Thus, the results obtained may be important to elucidate the adaptation of *S. epidermidis* strains within the host.

## 4. Materials and Methods

### 4.1. Ethics Statement

Human blood was collected into lithium heparin spray-coated tubes (VACUETTE^®^, Krems-münster, Austria) from healthy adult volunteers (both male and females, >18 years old) under a protocol approved by the Institutional Review Board of the University of Minho (SECVS 002/2014 (ADENDA)). Exclusion criteria included (i) any symptoms of infection at the time of blood collection or in the past week, (ii) antimicrobial therapy in the previous 14 days, and (iii) hospitalization in the last month. Furthermore, this procedure was performed in agreement with the Helsinki declaration and Oviedo convention and all donors gave written consent before blood collection.

### 4.2. Bacterial Strains

A worldwide collection of *S. epidermidis* strains/isolates was used in this study and is listed in Table S3 [16,22,23,24,25,26,27,28,29]. The criteria used to classify the origin of isolates (infection or contamination) from Denmark (DEN) and Iceland (ICE) are detailed in [16]. In brief, in the case of DEN isolates, (i) blood isolates wereclassified based on other possible infection sites in the same patients. For that, results obtained in additional samples such as urine, sputum, other blood cultures, abscess, and medical devices were also considered; in the case of (ii) urine samples, infection was considered when significant growth was detected in pure cultures; (iii) in the case of the respiratory tract samples, infection classification was based on the visualization of bacterial growth in the sputum under the microscope, (iv) for wounds, the isolates recovered from abscesses were considered to be from infection and the ones isolated from chronic ulcers were considered from colonization, and, finally, (v) in the case of samples collected from continuous ambulatory peritoneal dialysis devices, a significant growth from tips was classified as infection. For ICE isolates, (i) the presence of bacteria in more than one blood culture was regarded as infection, while a single positive blood culture was considered colonization; (ii) in urine samples, when >100,000 bacteria/mL was detected in relatively pure cultures, the isolates were classified as from infection and below that were regarded as commensals and, finally, (iii) in the case of wound samples, only when more than 15 colonies were detected in pure cultures of either open heart surgery wounds or wounds from catheter the isolates were considered from infection. All the remaining isolates were considered as being from infection or contamination, according to local clinicians’ evaluation criteria. In the case of the commensal isolates, these were collected and isolated from healthy volunteers from the Northern region of Portugal as previously described [27].

### 4.3. Identification of Potential Molecular Markers

#### 4.3.1. RNA Sequencing Analysis

Forty-eight hours old biofilms of the three clinical (IE214, PT12003, 1457) and three commensal (SECOM005A, SECOM020A.1, SECOM030) were formed in 24-well plates as previously described [30]. The cells in the 48-h biofilm bulk liquid were collected by centrifugation, suspended in 1 mL of 0.9% of NaCl, and sonicated for 10 s at 33% of amplitude to eliminate bacterial aggregates. Of note, the selected sonication cycle has no significant effect on cell viability, as previously determined by CFU counting and propidium iodide incorporation [31]. A suspension of 1 × 10^9^ CFU/mL was prepared, and, in 2 mL tubes, 100 μL of this suspension was mixed with 900 μL of human blood and incubated at 80 rpm for 2 h at 37 °C. Thereafter, RNA isolation, library preparation, and sequencing were performed as described before [32,33]. Briefly, bacterial cell lysis was achieved by mechanical (zirconium beads) and chemical (phenol) lysis as previously optimized [34], and total RNA was isolated using RNeasy Mini Kit (QIAGEN, Hilden, Germany). Since RNA integrity is essential to construct quality libraries, the Experion™ automated electrophoresis system (Bio-Rad, Hercules, CA, USA) was used to evaluate RNA integrity. Only samples with RNA quality indicators above nine were used for further analysis. To decrease the variability associated with each donor, RNA samples isolated from each of the three independent co-incubation assays performed were pooled together before library preparation. The samples were then treated with TURBO DNase (Ambion, MA, USA) and acid-phenol:chloroform:isoamyl alcohol (125:24:1) (Ambion) to degrade and isolate, respectively, contaminating genomic DNA. As bacteria were co-incubated with human blood cells, the kit MICROBEnrich™ kit (Ambion) was used to remove potentially contaminating eukaryotic RNA. Subsequently, bacterial messenger RNA was enriched by depleting ribosomal RNA using a Ribo-Zero™ rRNA removal kit for Gram-positive bacteria (Illumina, San Diego, CA, USA). Finally, using ScriptSeq™ RNA-seq library preparation kit (Illumina), the libraries were prepared, and their quality was assessed by quantitative PCR and Hi-Sensitivity D1K TapeStation (Agilent 2200 TapeStation). All libraries were then multiplexed, and sequencing data were generated from paired-end reads (2 × 150 bp) in a MiSeq^®^ sequencer (Illumina).

#### 4.3.2. Bioinformatic Analysis

CLC Genomics Workbench version 5.1 (QIAGEN) was used for quality, ambiguity, and length trimming, alignment with *S. epidermidis* RP62A (GenBank accession number: CP000029.1), and normalization of the reads per kilobase per million mapped reads (RPKM) [35]. Quality, ambiguity, and length trimming were performed using the CLC genomics workbench default settings. Raw and analyzed data sets are deposited in NCBI’s Gene Expression Omnibus database and are accessible through GEO series accession number GSE179407.

### 4.4. Validation of Candidate Diagnostic Markers

#### 4.4.1. PCR Detection of Genes with Potential Discriminatory Power

For gene detection, genomic DNA was isolated from one to five colonies, collected from TSA plates not older than two days, and suspended in 200 μL of nuclease-free water. To lyse the cells, bacterial suspensions were heated, at 95 °C, for 10 min and immediately cooled in ice for 5 min. The lysate was then collected by 5 min centrifugation, and 1 μL was used as the template for PCR amplification together with 0.5 µL of forward and reverse primers (10 µM/each), 5 µL of NZYTaq II Green Master mix (NZYTech, Lisboa, Portugal) and 3 µL of water. The amplification was completed with the subsequent cycling parameters: 5 min at 95 °C followed by 35 repeats of 30 s at 95 °C, 15 s at 58 °C or 60 °C and 45 s at 72 °C, with a final extension step of 10 min at 72 °C, in an MJ Mini thermal cycler (Bio-Rad, Hercules, CA, USA). PCR products were then separated and analyzed by electrophoresis in a 1% agarose gel (NZYTech) stained with Midori Green DNA stain (Nippon Genetics Europe GmbH, Düren, Germany) and visualized using a ChemiDoc™ XRS+ (Bio-Rad). The primers used were designed with the support of Primer3 software [36] using *S. epidermidis* RP62A as the template (Table S4).

#### 4.4.2. Co-Incubation of Bacteria and Blood

To reduce the use of human blood, for all validation studies, the co-incubation assays were performed in a smaller volume than the one used for RNA-seq analysis [37]. Defibrinated horse blood (Thermo Fisher Scientific, Waltham, MA, USA) was used to analyze gene expression in a larger collection of isolates. Briefly, 50 μL of each bacterial suspension with 1 × 10^9^ CFU/mL were mixed with 450 μL of either human or horse blood and incubated at 80 rpm and 37 °C for up to 6 h.

#### 4.4.3. Quantitative PCR (qPCR)

To validate the transcription levels of the selected genes, qPCR was performed as optimized before [34,37]. For biological validation of the data obtained by RNA-seq, total RNA was extracted using the E.Z.N.A.^®^ Total RNA Kit I (omega Bio-tec, Norcross, GA, USA). Thereafter, all RNA samples were treated with DNase I (Thermo Fisher Scientific), and RNA concentration and purity were determined using a NanoDrop^TM^ 1000. RNA integrity was evaluated by electrophoresis. Complementary DNA (cDNA) was synthesized, from 200 ng of total RNA, by RevertAid M-MuLV enzyme (Thermo Fisher Scientific) and using random primers as a priming strategy. The qPCR reaction was prepared using Xpert iFast SYBR Mastermix (Grisp, Porto, Portugal) following the manufacturer’s instructions. The run was completed in a CFX96^TM^ thermal cycler (Bio-Rad) with the following cycling parameters: 3 min at 95 °C followed by 39 cycles of 5 s at 95 °C and 25 s at 60 °C. The normalized expression was accomplished using 16S rRNA as a reference gene and by applying the delta Cq method (E^ΔCq^), where ΔCq = Cycle quantification (reference gene)—Cycle quantification (target gene) and E is the experimentally determined reaction efficiency. The primers used for qPCR were designed using Primer3 software [36], having *S. epidermidis* RP62A as the template (Table S4).

### 4.5. Statistical Analysis

Statistical analysis was carried out with GraphPad Prism, using either the unpaired *t*-test or one-way ANOVA with Tukey’s multiple comparison test, as described in the Figures caption. *p*-values less than 0.05 were considered significant.

## Data Availability

Raw and analyzed RNA sequencing data are available in a publicly accessible repository. The data presented in this study are openly available in [GEO, series accession number [GSE179407].

## Supplementary Materials

The following supporting information can be downloaded at: https://www.mdpi.com/article/10.3390/antibiotics11111596/s1, Figure S1: Experimental set-up workflow; Figure S2: RNA-seq in silico data analysis workflow; Figure S3: Transcription levels of the genes (a) *SERP0887*, (b) *SERP1064* and (c) *SERP2064* after 4 h of incubation in human or horse blood; Figure S4: Transcription levels of the genes (a) *SERP0887* (b) *SERP1064* and (c) *SERP2064* after 4 h of incubation in horse blood in a collection of *S. epidermidis* isolates; Table S1: List of candidates obtained using different in silico analysis strategies; Table S2: Prevalence of the genes with discriminatory potential in *S. epidermidis* isolates; Table S3: List of S. epidermidis isolates used in this study; Table S4: List of primers used for PCR and/or qPCR.

## References

[1] Otto M. (2009). *Staphylococcus epidermidis*—The “accidental” pathogen. Nat. Rev. Microbiol..

[2] Hogan S., Stevens N.T., Humphreys H., O’Gara J.P., O’Neill E. (2014). Current and future approaches to the prevention and treatment of staphylococcal medical device-related infections. Curr. Pharm. Des..

[3] Hall K.K., Lyman J.A. (2006). Updated review of blood culture contamination. Clin. Microbiol. Rev..

[4] Conlan S., Mijares L.A., Becker J., Blakesley R.W., Bouffard G.G., Brooks S., Coleman H., Gupta J., Gurson N., Park M. (2012). *Staphylococcus epidermidis* pan-genome sequence analysis reveals diversity of skin commensal and hospital infection-associated isolates. Genome Biol..

[5] Espadinha D., Sobral R.G., Mendes C.I., Méric G., Sheppard S.K., Carriço J.A., de Lencastre H., Miragaia M. (2019). Distinct phenotypic and genomic signatures underlie contrasting pathogenic potential of staphylococcus epidermidis clonal lineages. Front. Microbiol..

[6] Gu J., Li H., Li M., Vuong C., Otto M., Wen Y., Gao Q. (2005). Bacterial insertion sequence IS256 as a potential molecular marker to discriminate invasive strains from commensal strains of *Staphylococcus epidermidis*. J. Hosp. Infect..

[7] Cherifi S., Byl B., Deplano A., Nonhoff C., Denis O., Hallin M. (2013). Comparative epidemiology of *Staphylococcus epidermidis* isolates from patients with catheter-related bacteremia and from healthy volunteers. J. Clin. Microbiol..

[8] Salgueiro V.C., Iorio N.L.P., Ferreira M.C., Chamon R.C., Santos K.R.N. (2017). Methicillin resistance and virulence genes in invasive and nasal *Staphylococcus epidermidis* isolates from neonates. BMC Microbiol..

[9] Du X., Zhu Y., Song Y., Li T., Luo T., Sun G., Yang C., Cao C., Lu Y., Li M. (2013). Molecular analysis of *Staphylococcus epidermidis* strains isolated from community and hospital environments in China. PLoS ONE.

[10] Du X., Larsen J., Li M., Walter A., Slavetinsky C., Both A., Sanchez Carballo P.M., Stegger M., Lehmann E., Liu Y. (2021). *Staphylococcus epidermidis* clones express *Staphylococcus aureus*-type wall teichoic acid to shift from a commensal to pathogen lifestyle. Nat. Microbiol..

[11] VanAken S.M., Newton D., VanEpps J.S. (2021). Improved diagnostic prediction of the pathogenicity of bloodstream isolates of *Staphylococcus epidermidis*. PLoS ONE.

[12] Coenye T. (2021). Do results obtained with RNA-sequencing require independent verification?. Biofilm.

[13] Whitney A.R., Diehn M., Popper S.J., Alizadeh A.A., Boldrick J.C., Relman D.A., Brown P.O. (2003). Individuality and variation in gene expression patterns in human blood. Proc. Natl. Acad. Sci. USA.

[14] Cobb J.P., Mindrinos M.N., Miller-Graziano C., Calvano S.E., Baker H.V., Xiao W., Laudanski K., Brownstein B.H., Elson C.M., Hayden D.L. (2005). Application of genome-wide expression analysis to human health and disease. Proc. Natl. Acad. Sci. USA.

[15] Eady J.J., Wortley G.M., Wormstone Y.M., Hughes J.C., Astley S.B., Foxall R.J., Doleman J.F., Elliott R.M. (2005). Variation in gene expression profiles of peripheral blood mononuclear cells from healthy volunteers. Physiol. Genomics.

[16] Miragaia M., Thomas J.C., Couto I., Enright M.C., Lencastre H. (2007). Inferring a population structure for *Staphylococcus epidermidis* from multilocus sequence typing data. J. Bacteriol..

[17] França A., Cerca N. (2016). Plasma is the main regulator of *Staphylococcus epidermidis* biofilms virulence genes transcription in human blood. Pathog. Dis..

[18] Cau L., Williams M.R., Butcher A.M., Nakatsuji T., Kavanaugh J.S., Cheng J.Y., Shafiq F., Higbee K., Hata T.R., Horswill A.R. (2021). Staphylococcus epidermidis protease EcpA can be a deleterious component of the skin microbiome in atopic dermatitis. J. Allergy Clin. Immunol..

[19] Both A., Huang J., Qi M., Lausmann C., Weißelberg S., Büttner H., Lezius S., Failla A.V., Christner M., Stegger M. (2021). Distinct clonal lineages and within-host diversification shape invasive *Staphylococcus epidermidis* populations. PLoS Pathog..

[20] Teichmann P., Both A., Wolz C., Hornef M.W., Rohde H., Yazdi A.S., Burian M. (2022). The *Staphylococcus epidermidis* transcriptional profile during carriage. Front. Microbiol..

[21] Zhou W., Spoto M., Hardy R., Guan C., Fleming E., Larson P.J., Brown J.S., Oh J. (2020). Host-specific evolutionary and transmission dynamics shape the functional diversification of *Staphylococcus epidermidis* in human skin. Cell.

[22] Cerca N., Martins S., Sillankorva S., Jefferson K.K., Pier G.B., Oliveira R., Azeredo J. (2005). Effects of growth in the presence of subinhibitory concentrations of dicloxacillin on Staphylococcus epidermidis and Staphylococcus haemolyticus biofilms. Appl. Environ. Microbiol..

[23] Cerca N., Pier G.B., Vilanova M., Oliveira R., Azeredo J. (2004). Influence of batch or fed-batch growth on *Staphylococcus epidermidis* biofilm formation. Lett. Appl. Microbiol..

[24] Christensen G.D., Simpson W.A., Bisno A.L., Beachey E.H. (1982). Adherence of slime-producing strains of *Staphylococcus epidermidis* to smooth surfaces. Infect. Immun..

[25] Freitas A.I., Lopes N., Oliveira F., Brás S., França Â., Vasconcelos C., Vilanova M., Cerca N. (2018). Comparative analysis between biofilm formation and gene expression in *Staphylococcus epidermidis* isolates. Future Microbiol..

[26] Mack D., Siemssen N., Laufs R. (1992). Parallel induction by glucose of adherence and a polysaccharide antigen specific for plastic-adherent *Staphylococcus epidermidis*: Evidence for functional relation to intercellular adhesion. Infect. Immun..

[27] Oliveira F., Cerca N. (2013). Antibiotic resistance and biofilm formation ability among coagulase-negative staphylococci in healthy individuals from portugal. J. Antibiot..

[28] Gill S.R., Fouts D.E., Archer G.L., Mongodin E.F., DeBoy R.T., Ravel J., Paulsen I.T., Kolonay J.F., Brinkac L., Beanan M. (2005). Insights on evolution of virulence and resistance from the complete genome analysis of an early methicillin-resistant Staphylococcus aureus strain and a biofilm-producing methicillin-resistant Staphylococcus epidermidis strain. J. Bacteriol..

[29] Cerca N., Gomes F., Bento J.C., França A., Rolo J., Miragaia M., Teixeira P., Oliveira R. (2013). Farnesol induces cell detachment from established *S. epidermidis* biofilms. J. Antibiot..

[30] França A., Carvalhais V., Vilanova M., Pier G.B., Cerca N. (2016). Characterization of an in vitro fed-batch model to obtain cells released from *S. epidermidis* biofilms. AMB Express.

[31] Freitas A.I., Vasconcelos C., Vilanova M., Cerca N. (2014). Optimization of an automatic counting system for the quantification of *Staphylococcus epidermidis* cells in biofilms. J. Basic Microbiol..

[32] França A., Pier G.B., Vilanova M., Cerca N. (2016). Transcriptomic analysis of *Staphylococcus epidermidis* biofilm-released cells upon interaction with human blood circulating immune cells and soluble factors. Front. Microbiol..

[33] França A., Carvalhais V., Maira-Litrán T., Vilanova M., Cerca N., Pier G. (2014). Alterations in the *Staphylococcus epidermidis* biofilm transcriptome following interaction with whole human blood. Pathog. Dis..

[34] França A., Freitas A.I., Henriques A.F., Cerca N. (2012). Optimizing a qPCR gene expression quantification assay for *S. epidermidis biofilms*: A comparison between commercial kits and a customized protocol. PLoS ONE.

[35] Mortazavi A., Williams B., McCue K., Schaeffer L., Wold B. (2008). Mapping and quantifying mammalian transcriptomes by RNA-Seq. Nat. Methods.

[36] Untergasser A., Cutcutache I., Koressaar T., Ye J., Faircloth B.C., Remm M., Rozen S.G. (2012). Primer3-new capabilities and interfaces. Nucleic Acids Res..

[37] Brás S., França A., Cerca N. (2020). Optimizing a reliable ex vivo human blood model to analyze expression of *Staphylococcus epidermidis* genes. PeerJ.

