# Supplementary material for: Transcriptome Mining to Identify Molecular Markers for the Diagnosis of Staphylococcus epidermidis Bloodstream Infections
Source: Antibiotics (Basel). 2022 Nov 11;11(11):1596. doi: 10.3390/antibiotics11111596 (PMC9687011; doi:10.3390/antibiotics11111596)
Supplement: Supplementary file 1 [file antibiotics-11-01596-s001.zip › antibiotics-1907157-supplementary.pdf]

Supplementary materials for:

Transcriptome mining to identify molecular markers for the diagnosis of *Staphylococcus epidermidis* bloodstream infections

Susana Brás [1] and Angela França [1,2,*]

[1] LIBRO – Laboratório de Investigação em Biofilmes Rosário Oliveira, Centre of Biological Engineering, University of Minho, Campus de Gualtar, 4710-057, Braga, Portugal
[2] LABBELS –Associate Laboratory, Braga and Guimarães, Portugal
* Correspondence: afranca@ceb.uminho.pt

Supplementary Figures

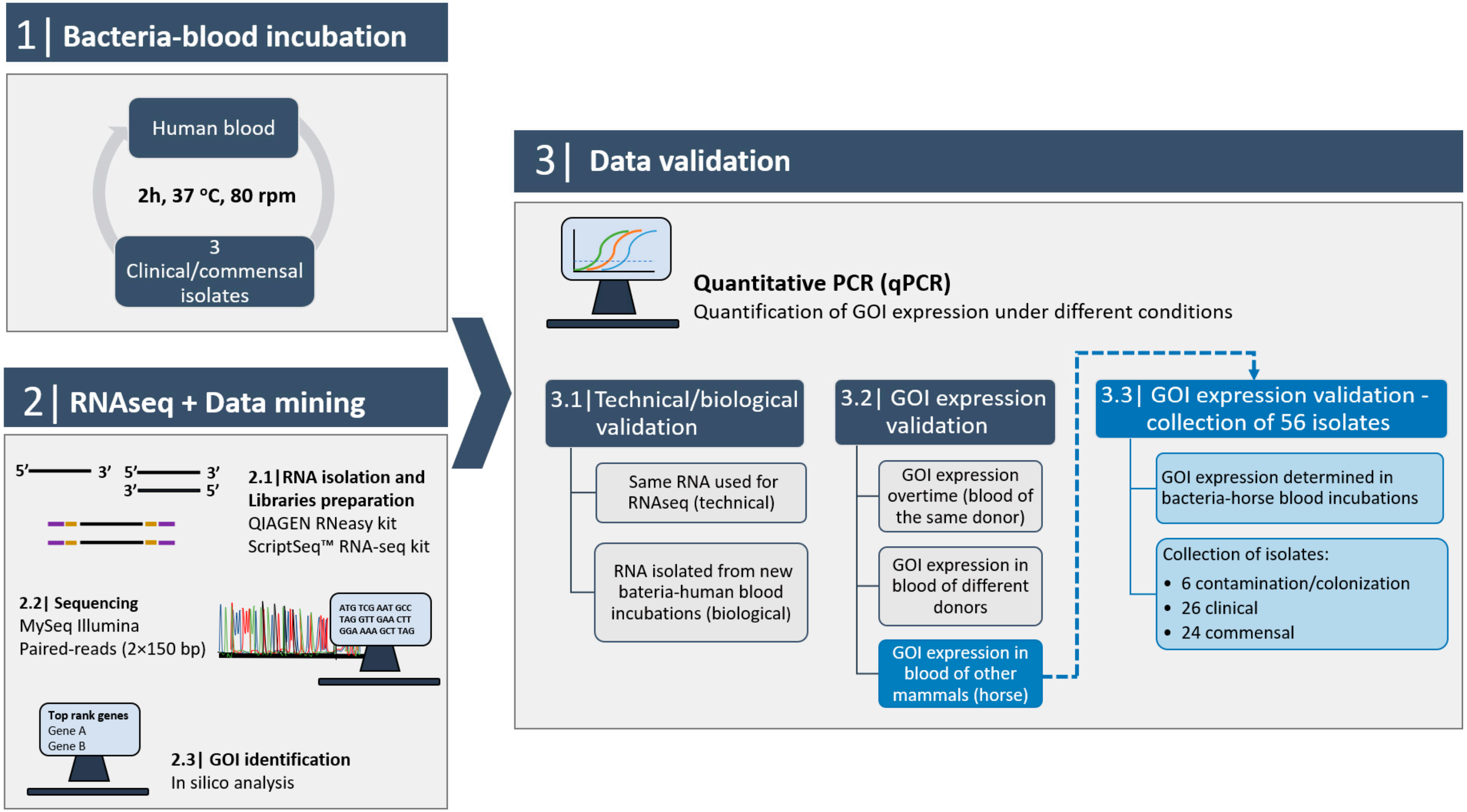

Figure S1. Experimental set-up workflow. RNA-seq, RNA sequencing; GOI, genes of interest.

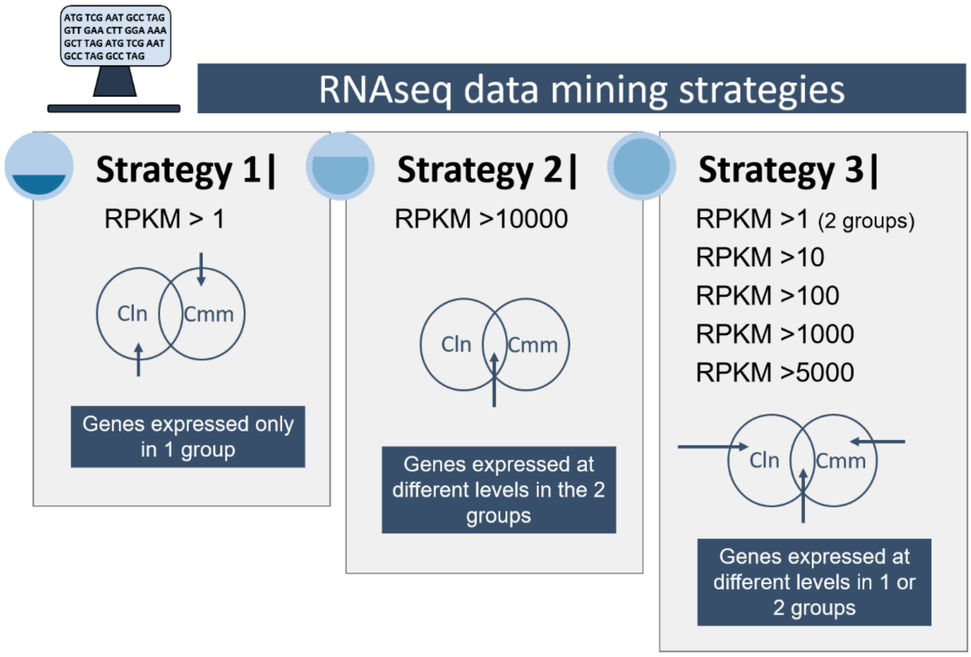

Figure S2. RNA-seq in silico data analysis workflow. Bioinformatics analysis based on RPKM thresholds was performed using three strategies. The first strategy (1) identified genes only detected in either clinical or commensal isolates. The second (2) and third (3) strategies were performed to identify differentially expressed genes using different RPKM thresholds (RPKM >1, RPKM >10, RPKM >100, RPKM >1000, RPKM >5000 and RPKM > 10000). Cln, clinical isolates; Cmm, commensal isolates; RPKM, reads per million mapped read

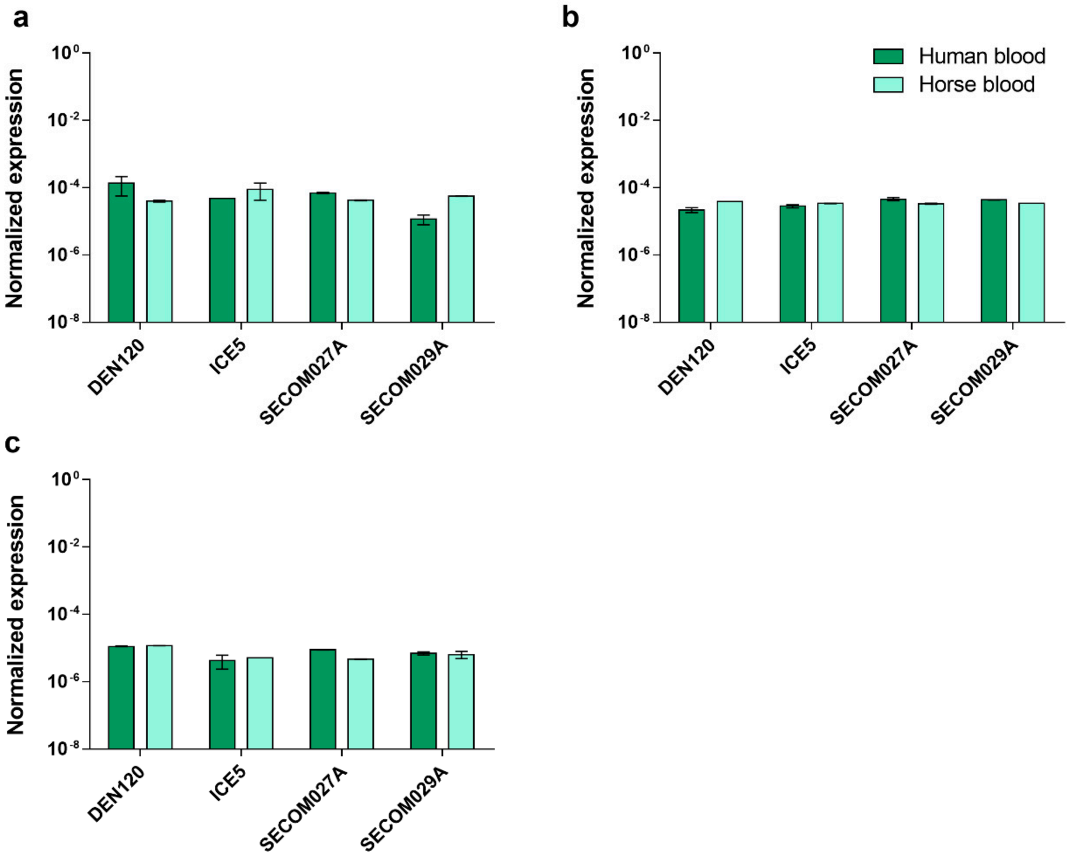

Figure S3. Transcription levels of the genes (a) *SERP0887*, (b) *SERP1064* and (c) *SERP2064* after 4 h of incubation in human or horse blood. These experiments were performed in two clinical (ICE5, DEN120) and two commensal (SECOM027A, SECOM029A) isolates. The bars represent the mean and standard error of the mean of two technical replicates. Statistical differences between groups were analyzed using the unpaired t-test.

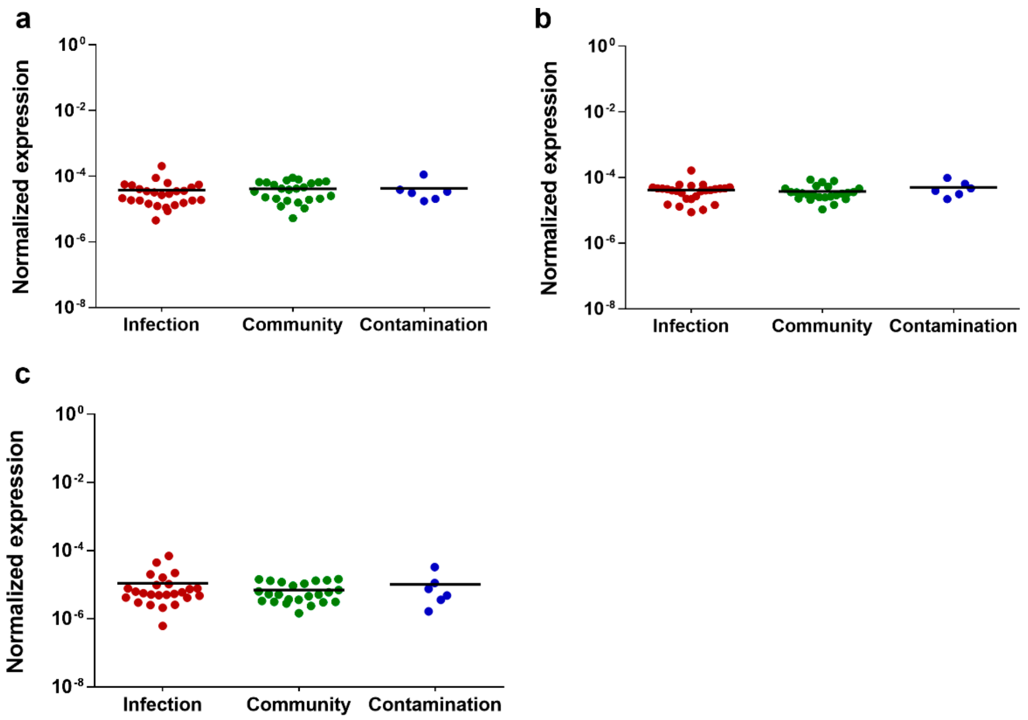

Figure S4. Transcription levels of the genes (a) *SERP0887* (b) *SERP1064* and (c) *SERP2064* after 4 h of incubation in horse blood in a wide collection of *S. epidermidis* isolates. The horizontal line represents the grand mean of the transcription levels in all isolates (n=2 to 3 technical qPCR replicates). Statistical differences among groups were analyzed with one-way ANOVA and Tukey´s multiple comparison test.

Supplementary tables

Table S1. List of candidates obtained using different in silico analysis strategies. KPKM, reads per million mapped reads; SEM, Standard error of the mean.

Gene	Function or putative function	RPKM		Fold-change (Clinical/Commensal)
		Clinical isolates (mean ± SEM)	Commensal isolates (mean ± SEM)	
SERP0012	Transcriptional regulator, Cro/CI family	11.56 ± 1.13	5.63 ± 5.02	2.05
SERP0886	ABC transporter, ATP-binding protein	48.54 ± 35.29	7.18 ± 3.47	6.76
SERP0887	ABC transporter, permease protein	52.62 ± 29.28	5.44 ± 3.43	9.67
SERP0888	Sensor histidine kinase, putative	39.07 ± 10.92	8.71 ± 2.46	4.48
SERP1064	Oxidoreductase, short-chain dehydrogenase/reductase family	65.31 ± 13.12	23.82 ± 9.59	2.74
SERP2064	PAP2 family protein	31.47± 11.61	6.52 ± 2.28	4.83
SERP2220	Universal stress protein family	9.58 ± 2.44	82.16 ± 27.14	- 8.33
SERP2255	Uncharacterized protein	14.60 ± 4.69	3.87 ± 0.80	3.77
SERP2397	Phenol soluble modulin beta 1	4.53 ± 3.93	28.16 ± 14.01	- 6.25
SERP2441	SLC45 family MFS transporter	0.18 ± 0.31	133.63 ± 46.20	- 769.23

Table S2. Prevalence of the genes with discriminatory potential in *S. epidermidis* isolates. The presence or absence of the genes was determined by PCR.

Genes	Clinical isolates (38 in total)	Commensal isolates (24 in total)	Contaminating isolates (6 in total)
SERP2441	26 (68%)	24 (100%)	6 (100%)
SERP0887	38 (100%)	24 (100%)	6 (100%)
SERP1064	38 (100%)	24 (100%)	6 (100%)
SERP2064	38 (100%)	24 (100%)	6 (100%)

Table S3. List of *S. epidermidis* isolates used in this study. M, male; F, female; SCCmec, Staphylococcal Cassette Chromosome mec, ST, Sequencing Type; CC, Clonal Complex; MSSE, Methicillin Susceptible *Staphylococcus epider*midis; NR, no record.

Strain/isolate	Country	Year	Gender	Age	Origin	Source	SCCmec, ST, CC	Reference
**Clinical isolates**								
**PT11002**	Portugal	2011	M	5	Infection	Blood	Unknown	[25]
**PT11015**	Portugal	2011	F	25	Infection	Blood	Unknown	
**PT12023**	Portugal	2012	M	69	Infection	Blood	Unknown	
**PT12030**	Portugal	2012	M	<1	Infection	Blood	Unknown	
**PT12032**	Portugal	2012	F	66	Infection	Blood	Unknown	
**PT12050**	Portugal	2012	M	56	Infection	Blood	Unknown	
**PT12060**	Portugal	2012	M	50	Infection	Blood	Unknown	
**PT12065**	Portugal	2012	M	75	Infection	Blood	Unknown	
**PT13042**	Portugal	2013	M	43	Infection	Blood	Unknown	
**PT13011**	Portugal	2013	NR	NR	Infection	Blood	Unknown	
**PT13038**	Portugal	2013	F	75	Infection	Blood	Unknown	
**ESP43**	Spain	1997	F	59	Infection	Blood	SCCmec III	[16]
**RP62A**	USA	1979	NR	NR	Infection	Blood	II, 10, 2	[24,28]
**MCO150**	Mexico	1998	M	6	Infection	Blood	IV, 46, 2	[16]
**MEX60**	Mexico	1996	NR	<12	Infection	Catheter	NT/2, 61, 2	
**PT12008**	Portugal	2012	F	65	Infection	Catheter	Unknown	[25]
**1457**	Germany	1989	NR	NR	Infection	Central Venous Catheter	MSSE, 86, 2	[26,29]
**MEX37**	Mexico	1996	NR	NR	Infection	Cerebrospinal fluid	II, 71, 11	[16]
**IE186**	USA	NR	NR	NR	Infection	Endocarditis	IV, 367, S367	[22,29]
**IE214**	USA	NR	NR	NR	Infection	Endocarditis	NT, 10, 2	
**TAW113**	Taiwan	1997	M	76	Infection	Respiratory tract	MS, 85, 2	[16]
**URU23**	Uruguay	1997	M	37	Infection	Urine	IV, 86, 2	
**ICE5**	Argentina	1997	M	79	Infection	Urine	IV, 23, 2	
**ICE9**	Iceland	1997	F	68	Infection	Urine	III, 6, 2	
**COB17**	Colombia	1997	NR	NR	Infection	Urine	33, 33, 33	
**COB20**	Colombia	1997	NR	<1	Infection	Urine	SCCmec IV	
**HUR51**	Hungary	1997	M	44	Infection	Wound	B/3, 47, 33	
**ICE102**	Iceland	1998	M	76	Infection	Wound	IV, 52, 2	
**ICE21**	Iceland	1997	F	57	Infection	Wound	I, 36, 2	
**ICE24**	Iceland	1997	M	76	Infection	Wound	IV, 38, 2	
**ITL34**	Italy	1997	M	41	Infection	Wound	IV, 66, 66	

**PLN64**	Poland	1997	M	50	Infection	Wound	NT/2, 64, 247	
**DEN19**	Denmark	1997	M	76	Infection	NR	IV, 1, 2	
**DEN120**	Denmark	1998	M	27	Infection	NR	A/C, 40, 2	
**GRE26**	Greece	1998	NR	NR	Infection	NR	IV, 11, 11	
**PT12003**	Portugal	2012	M	62	Surgical Site Infection	Central catheter	Unknown	[25]
**PT12005**	Portugal	2012	M	75	Surgical Site Infection	Blood	Unknown	
**PT12013**	Portugal	2012	M	90	Respiratory Tract Infection	Blood	Unknown	
**Contamination/colonization isolate**								
**PE9**	Boston	NR	NR	NR	Colonization	NR	II, 10, 2	[23, 29]
**DEN110**	Denmark	1998	M	50	Colonization	NR	IV, 68, 66	[26]
**CV45**	Cape Verde	1997	F	10	Colonization	NR	IV, 79, 2	
**DEN185**	Denmark	1998	M	41	Contamination	Blood	IV, 21, 2	
**DEN69**	Denmark	1997	F	35	Contamination	Blood	V, 56, S56	
**ICE192**	Iceland	1998	M	2	Contamination	Blood	IV, 5, 2	
**Commensal isolates**								
**SECOM005A**	Portugal	2012	F	23	Skin	Skin	Unknown	[27]
**SECOM020A.1**	Portugal	2012	M	15	Skin	Skin	Unknown	
**SECOM030A**	Portugal	2012	F	45	Skin	Skin	Unknown	
**SECOM001B**	Portugal	2012	F	56	Skin	Skin	Unknown	
**SECOM003A**	Portugal	2012	M	35	Skin	Skin	Unknown	
**SECOM010B**	Portugal	2012	M	52	Skin	Skin	Unknown	
**SECOM022A**	Portugal	2012	F	14	Skin	Skin	Unknown	
**SECOM023A**	Portugal	2013	M	22	Skin	Skin	Unknown	
**SECOM024A**	Portugal	2013	M	17	Skin	Skin	Unknown	
**SECOM027A**	Portugal	2013	F	48	Skin	Skin	Unknown	
**SECOM029A**	Portugal	2013	M	50	Skin	Skin	Unknown	
**SECOM031A**	Portugal	2013	M	25	Skin	Skin	Unknown	
**SECOM034A**	Portugal	2013	M	19	Skin	Skin	Unknown	
**SECOM035A**	Portugal	2013	M	20	Skin	Skin	Unknown	
**SECOM037A**	Portugal	2013	M	19	Skin	Skin	Unknown	
**SECOM040A**	Portugal	2013	F	19	Skin	Skin	Unknown	
**SECOM042A**	Portugal	2013	F	20	Skin	Skin	Unknown	
**SECOM049A**	Portugal	2013	M	28	Skin	Skin	Unknown	
**SECOM053A**	Portugal	2013	M	27	Skin	Skin	Unknown	
**SECOM058A**	Portugal	2013	M	48	Skin	Skin	Unknown	
**SECOM062A**	Portugal	2013	M	10	Skin	Skin	Unknown	

**SECOM066A**	Portugal	2013	F	19	Skin	Skin	Unknown
**SECOMF12**	Portugal	2013	F	21	Skin	Skin	Unknown
**SECOMM14**	Portugal	2013	M	21	Skin	Skin	Unknown

Table S4. List of primers used for PCR and /or qPCR. bp, base pair

Gene	Primer sequence (5´-3′)	Amplicon (bp)	Efficiency (%)
16S rRNA	Fw: GGGCTACACACGTGCTACAA<br>Rv: GTACAAGACCCGGGAACGTA	176	97
SERP0012	Fw: TCACAAGAATTTTTGGCTGAG<br>Rv: TTGATTTGCACGTTTTTCAA	167	98
SERP0886	Fw: GAGCCTACGTCAGCTATGGA<br>Rv: TTGATCAGCCATTCTTTCAAC	132	110
SERP0887	Fw: TTTCCTGATTGGCTTCAGTC<br>Rv: GCCAAACGCTTCTATATCCA	108	107
SERP0888	Fw: AAAGTGCGGCTAAATCATTAAA<br>Rv: TGATGCTCATGTACCTCCAA	133	108
SERP1064	Fw: AATGTACAGCCCGGTCCAATAGAT<br>Rv: TATTTGCTTGGCAAGTTGTTGAGG	117	99
SERP2064	Fw: ATTCGTGTCAGAAACCGCTCAA<br>Rv: GCATTTCCAGCGTTTCCTTCAT	102	91
SERP2220	Fw: ATGATTTTAGTGCTATCCCTGACT<br>Rv: CACTAATTGCAAGATCATTTTCT	102	111
SERP2255	Fw: CACAATCAATTCCCGCAAGT<br>Rv: GACGGCATCACCACTTCCTA	182	108
SERP2397	Fw: ATAACCAAATGCGTGAGCAA<br>Rv: ACAGCAGCACAAGCAGAAGA	105	94
SERP2441	Fw: CAGGCATTGAACTTCCCAAT<br>Rv: AATTCGGGGGCATATTTAGG	109	103
